# Genomics of Clinal Local Adaptation in *Pinus sylvestris* Under Continuous Environmental and Spatial Genetic Setting

**DOI:** 10.1534/g3.120.401285

**Published:** 2020-06-16

**Authors:** Jaakko S. Tyrmi, Jaana Vuosku, Juan J. Acosta, Zhen Li, Lieven Sterck, Maria T. Cervera, Outi Savolainen, Tanja Pyhäjärvi

**Affiliations:** * Department of Ecology and Genetics, University of Oulu, FI-90014 Oulu, Finland,; ^†^ Biocenter Oulu, University of Oulu, FI-90014 Oulu, Finland,; ^‡^ Camcore, Department of Forestry and Environmental Resources, North Carolina State University, Raleigh, NC,; ^§^ Department of Plant Biotechnology and Bioinformatics, Ghent University, Technologiepark 71, 9052 Ghent, Belgium,; ^**^ VIB Center for Plant Systems Biology, Technologiepark 71, 9052 Ghent, Belgium, and; ^††^ Centro de Investigación Forestal (CIFOR), Instituto Nacional de Investigaciones Agrarias (INIA), 28040 Madrid, Spain

**Keywords:** adaptation, population genetics – empirical, landscape genetics, gymnosperms, *Pinus sylvestris*, Local adaptation, Targeted DNA Sequencing, Structural Variation

## Abstract

Understanding the consequences of local adaptation at the genomic diversity is a central goal in evolutionary genetics of natural populations. In species with large continuous geographical distributions the phenotypic signal of local adaptation is frequently clear, but the genetic basis often remains elusive. We examined the patterns of genetic diversity in *Pinus sylvestris*, a keystone species in many Eurasian ecosystems with a huge distribution range and decades of forestry research showing that it is locally adapted to the vast range of environmental conditions. Making *P. sylvestris* an even more attractive subject of local adaptation study, population structure has been shown to be weak previously and in this study. However, little is known about the molecular genetic basis of adaptation, as the massive size of gymnosperm genomes has prevented large scale genomic surveys. We generated a both geographically and genomically extensive dataset using a targeted sequencing approach. By applying divergence-based and landscape genomics methods we identified several loci contributing to local adaptation, but only few with large allele frequency changes across latitude. We also discovered a very large (ca. 300 Mbp) putative inversion potentially under selection, which to our knowledge is the first such discovery in conifers. Our results call for more detailed analysis of structural variation in relation to genomic basis of local adaptation, emphasize the lack of large effect loci contributing to local adaptation in the coding regions and thus point out the need for more attention toward multi-locus analysis of polygenic adaptation.

Populations of species with vast continuous distributions can inhabit very different environments. These populations are often locally adapted, defined as each population having higher fitness than any introduced population at its home site (Kawecki and Ebert 2004), preferably demonstrated by performing reciprocal transplant experiment (Savolainen *et al.* 2013) as has been done with many plant species, such as the *Arabidopsis* genus (*e.g.*, Leinonen *et al.* 2011; Ågren and Schemske 2012; Hämälä *et al.* 2018). Local adaptation can also be inferred from patterns of phenotypic variation or environmental correlation, as has been shown for example, in *Drosophila melanogaster* (Adrion *et al.* 2015), humans (Fan *et al.* 2016) and also forest trees (Giertych 1991; Mimura and Aitken 2007; Savolainen *et al.* 2007; Alberto *et al.* 2013b; Prunier *et al.* 2016; Gárate-Escamilla *et al.* 2019; Pyhäjärvi *et al.* 2020).

Local adaptation with a polygenic basis has received more attention lately, because a great deal of adaptive variation is quantitative with multiple underlying loci (Buckler *et al.* 2009; Rockman 2012; Berg and Coop 2014; Yeaman 2015; Hall *et al.* 2016; Boyle *et al.* 2017), also in forest trees (Lind *et al.* 2018). A well-known model of polygenic adaptation of a single population in a new environment is presented by the Fisher/Orr model (Fisher 1918; Orr 1998) which predicts an exponential distribution of QTL effects (see Barton & Keightley 2002). However, local adaptation arises due to differential selection in different populations in variable environments, possibly connected by gene flow. This kind of selection often results in phenotypic clines (Huxley 1938). Several theoretical predictions for the underlying genetic architecture of clines have been proposed. While differential selection along environmental gradients in continuous populations on single locus governed traits is expected to result in allele frequency clines (*e.g.*, Slatkin 1973), for polygenic models the expectations are more complex.

Barton (1999) has examined a model with polygenic architecture where a subset of loci will have successive, sharp allele frequency clines along the environmental gradient and maintain the phenotypic mean close to the optimum. The underlying loci are, perhaps unrealistically, expected to have similar effect size on the trait. Such sharp allele frequency clines would be seen as F_ST_ outliers, although the majority of loci governing the underlying traits may remain undetected as most alleles are expected to stay near fixation throughout the range in this model.

Latta (1998, 2003) and LeCorre and Kremer (2003, 2012; Kremer and Le Corre 2012) show that in a high gene flow and strong selection scenario of a polygenic trait, the contribution of covariance between loci becomes more important than between population allele frequency differentiation. Also, in a simulation study Yeaman (2015) showed that local adaptation is indeed a possible outcome even when only small effect alleles are present given that there is enough standing genetic variation. Most importantly in this model, the contributions of individual loci may be transient making detection of the contributing loci more difficult. Nonetheless, allele frequency clines have been observed in many empirical studies (*e.g.*, Schmidt *et al.* 2008; Adrion *et al.* 2015; Wang *et al.* 2018).

Even with genome-wide datasets of tens or hundreds of thousands of loci sampled across localities, identifying the loci underlying adaptive clinal variation remain a challenge. The majority of methods for uncovering adaptive loci are based on the island model of population structure (Lewontin and Krakauer 1973; Foll and Gaggiotti 2008; Excoffier *et al.* 2009; Vitalis *et al.* 2014) and do not fully utilize the spatial information on the clinal genetic variation. Environmental association analysis (Coop *et al.* 2010) and simple regression models can be used to identify clinal trends (Ma *et al.* 2010; Chen *et al.* 2012; Kujala *et al.* 2017).

It is also important to consider putative effects of recombination across adaptive loci on the genetic architecture of local adaptation. The effect of gene flow is expected to override the effect of weak differential selection on a particular locus. However, physical linkage between multiple small effect alleles makes them behave like a single large effect allele, as described by Yeaman and Whitlock (2011). They show that local adaptation under gene flow may favor genetic architecture where recombination is reduced between loci contributing to local adaptation, which may be caused by physical proximity, transposable element action, translocations or inversions (Kirkpatrick and Barton 2006). This will result in increased linkage disequilibrium (LD), and thus the examination of unusual LD patterns may be a fruitful approach in discovering the genetic architecture of local adaptation.

*Pinus sylvestris* (Scots pine) is a widely distributed conifer species with nearly three centuries of history as a study subject of forestry, ecology and adaptive variation (Pyhäjärvi *et al.* 2020). The range of *P. sylvestris* spans a huge distribution area in Eurasia from southern Spain to northern Scandinavia and eastern Russia. Its distribution is mostly continuous displaying only limited population structure in the nuclear genome, with the exception of some of the more isolated populations for instance in Spain and Italy (Karhu *et al.* 1996; Pyhäjärvi *et al.* 2007; Kujala and Savolainen 2012). However, differentiation within the main range can be seen in mitochondrial haplotype structure, providing information about the recent colonization routes of *P. sylvestris* (Cheddadi *et al.* 2006; Naydenov *et al.* 2007; Pyhäjärvi *et al.* 2008).

In *P. sylvestris* multiple latitudinal phenotypic clines have been repeatedly observed in traits important for abiotic adaptation such as cold tolerance (Eiche 1966; Aho 1994; Hurme *et al.* 1997, 2000) and the timing of growth start and cessation (Mikola 1982; Beuker 1994; Karhu *et al.* 1996). These traits vary latitudinally with environmental conditions, such as temperature, day length, UV radiation intensity and seasonality. Common garden experiments have shown that these traits have a considerable genetic basis suggesting local adaptation (Giertych 1991; Savolainen *et al.* 2007; Alberto *et al.* 2013a; Pyhäjärvi *et al.* 2020). When searching for genomic basis of local adaptation, demographic effects may lead to spurious signals if the underlying population structure remains unaccounted for (Hoban *et al.* 2016). Lack of genome-wide structure, together with highly differentiated phenotypic variation, makes *P. sylvestris* an ideal species for investigating the genetic basis of local adaptation in a large genome. Furthermore, the low level of LD (Wachowiak *et al.* 2009) and lack of any known hybridization with other species should aid in detecting non-equilibrium patterns in the genome. The genetic basis of the adaptation remains largely unknown even though some details have been uncovered in previous studies using data from few candidate genes. Only few F_ST_ outliers have been found, but several cases of latitudinal allele frequency clines and variants associated to timing of bud set have been uncovered (Kujala and Savolainen 2012; Kujala *et al.* 2017). Similar observations of allele frequency clines have been made in other tree species as well, such as *Populus* (Ma *et al.* 2010; Evans *et al.* 2014) and *Picea* (Holliday *et al.* 2010; Chen *et al.* 2012). However, many of these important traits likely have polygenic architecture, possibly complicating efforts in detecting the underlying genetic variation (Lind *et al.* 2018).

Clinal variation, genetic differences across the range and the effect of natural selection in *P. sylvestris* are obvious at the phenotypic level. In this study we create the first genome-wide dataset of *P. sylvestris* to examine patterns of genetic diversity and to search for genomic signature of local adaptation. Similarly to e.g. Yeaman *et al.* (2016), the use of exome capture instead of whole genome sequencing makes it feasible to sequence large number of samples in this species with a genome size of 23.6 Gbp (Zonneveld 2012), or roughly 7 times larger than the human genome. The use of exome capture allows the examination of a significant portion of the coding sequence for genetic diversity, long range LD patterns and the detection of large structural variants. However, this kind of data are not well suited – especially in *P. sylvestris* with limited genomic resources – for sliding window type of analysis or comparing features of coding, non-coding and intronic areas. In this study we use this novel data set sampled from wide geographical area to answer the following questions regarding the manifestation of the phenotypic patterns at the molecular level of variation: 1) Is the commonly applied discrete island population model properly describing the distribution of genetic diversity, or is a model incorporating continuous isolation-by-distance more suitable for a widely distributed and wind dispersed species such as *P. sylvestris*? 2) Is the strong local adaptation at the phenotypic level reflected in the genetic diversity as corresponding allele frequency clines, high differentiation among populations or increased LD in vicinity of selected sites in *P. sylvestris*?

## Materials And Methods

### Plant material and genotyping

**Figure 1:**
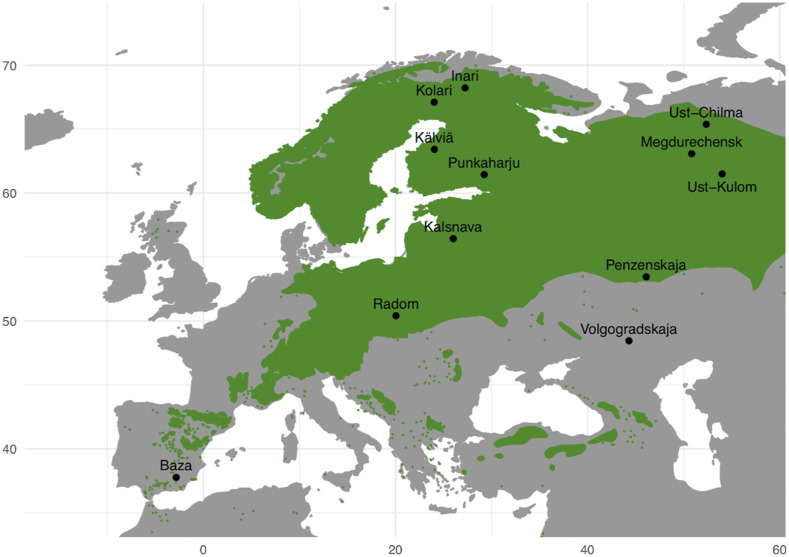
Map of sampling locations with *P. sylvestris* distribution is marked in green color.

Seeds from 109 *P. sylvestris* samples from 12 populations spanning 31 degrees of latitude were used in generating the dataset for this study (Figure 1, Table 1). The main sampling area included two latitudinal gradients, one from northern Finland to Poland and another north-south gradient in western Russia, to increase power of genome scans (Lotterhos and Whitlock 2015). A total of 120 samples were initially genotyped, of which one was later removed due to sampling the same tree twice and additional 10 were removed due to low sequencing coverage. Haploid genomic DNA was extracted from megagametophyte tissue by using E.Z.N.A. SP Plant DNA kit (Omega Biotek). DNA was fragmented to an average length of 200 nucleotides with Bioruptor ultrasonicator (Diagenode). Libraries were prepared by using NEBNext DNA Library Prep Master Mix Set for Illumina and NEBNext Multiplex Oligos for Illumina E7600S (New England BioLabs) to multiplex libraries of four samples. Targeted capture was performed for each pool according to MycroArray MYbaits protocol v.2.3.1.

**Table 1:** Study population location and summary statistic information

Population	Latitude	Longitude	$\pi_{4}$ ($\times 10^{-3}$)	$\pi_{0}/\pi_{4}$	Tajima’s D	Ρ ($\times 10^{-3}$)
Inari	68° 54’ N	27° 1’ E	3.90	0.404	−0.250	1.63
Kolari	67° 10’ N	24° 3′ E	3.71	0.393	−0.290	1.81
Kälviä	63° 51’ N	23° 27’ E	3.94	0.395	−0.259	1.49
Punkaharju	61° 45’ N	29° 23’ E	3.87	0.395	−0.283	2.27
Kalsnava	56° 43’ N	26° 1’ E	3.79	0.384	−0.299	1.21
Radom	50° 24’ N	20° 3′ E	3.85	0.400	−0.290	1.47
Ust-Chilma	65° 22’ N	52° 21’ E	3.94	0.396	−0.223	1.60
Megdurechensk	63° 4’ N	50° 49’ E	4.04	0.397	−0.283	1.81
Ust-Kulom	61° 30’ N	54° 0’ E	3.76	0.380	−0.301	1.19
Penzenskaja	53° 27’ N	46° 6’ E	4.12	0.398	−0.272	2.16
Volgogradskaja	47° 45’ N	44° 30’ E	4.19	0.400	−0.278	1.89
Baza	37° 46’ N	2° 49’ W	3.82	0.389	−0.156	1.11

### Bait design for targeted sequence capture

Bait design was based on a set of *P. sylvestris* transcripts described previously (Li *et al.* 2017). Briefly, transcriptomes of *P. sylvestris* were assembled from 454 read data derived from different developmental stages using the Newbler software (v2.8.1). Those were then integrated with public transcriptomes from PlantGDB-assembled Unique Transcripts (based on GenBank release 187) and a public set of EST assemblies. This initial set contained 121,538 transcripts on which 36,106 open reading frames (ORFs) were predicted by TransDecoder (r20131117). The ORFs were then mapped against the repeat masked *P. taeda* reference genome version 1.01 (Neale *et al.* 2014) with gmap (Wu and Nacu 2010) in order to obtain exon sequences. An ORF was omitted if it could be mapped equally well to several locations of the reference suggesting a paralogous sequence. In total 10,330 ORFs encompassing an area of 12,221,835 bp were selected as targets for initial bait design.

MycroArray MYbaits (Ann Arbor, MI) service was used to create an initial set of 100 base long baits with 2x tiling resulting in a total of 176,334 baits. Four pilot experiments including target capture and sequencing were then conducted to determine bait performance. The putative position of each bait in the genome was determined by aligning the bait sequences to the unmasked *P. taeda* reference genome v. 1.01 (Neale *et al.* 2014) with blastn. A well working bait was defined as having a unique high-quality hit to omit possible paralogous sequences, at least 75 out of 100 bases aligning to omit baits on exon-intron boundaries and less than 4% mismatches to ensure successful alignment. To analyze bait ability to capture target areas 2 × 100 bp paired-end sequencing reads were generated in total for 32 *P. sylvestris* megagametophyte and needle samples with Illumina Hiseq 2500 instrument and 2 × 150 bp paired-end reads with MiSeq instrument. Baits failing to capture any sequence were omitted from the final bait set. After filtering, 60,000 high quality bait sequences were selected as the final bait set (File S1) that was used for examining the 109 samples used in this study. Sequencing was performed using Illumina HiSeq 2500 instrument at Institute of Molecular Medicine Finland (FIMM), by multiplexing four randomly selected samples to each lane, with 100-bp paired-end reads

### Genotype calling workflow

Raw reads from Illumina sequencing were aligned to unmasked *P. taeda* reference genome version 1.01 with bowtie2 version 1.1.1 (Langmead and Salzberg 2012) using parameters to include only properly paired alignments (–no-mixed) omit discordant alignments (–no-discordant), and to omit results with no proper alignment (–no-unal). The resulting SAM files were modified with Picard toolkit (http://broadinstitute.github.io/picard/) and SAMtools (Li *et al.* 2009) by converting SAM files to BAM format with SamFormatConverter, sorting with SortSam, removing duplicate sequences with MarkDuplicates, defining read groups with AddOrReplaceReadGroups and indexing with SAMtools index. Examination of alignments revealed that despite omitting targets at known paralogous areas in the bait design stage, many baits captured paralogous sequences from unknown areas not covered in the *P. taeda* reference genome. This was causing issues in read alignment and often leading to spurious SNP calls. The process of detecting the issue and circumventing incorrect SNP calls is described in supplementary methods (File S2). In short, the SNP calling was performed twice using freebayes, first to detect problematic areas identified as heterozygous SNP calls not expected when sequencing haploid DNA, and second time to call SNPs only in problem-free areas.

The technical quality was evaluated by generating a fastqc quality report for raw reads, SAMtools flagstat report for alignment success, along with visual inspection of alignments with SAMtools tview and Integrative Genomics Viewer (Thorvaldsdóttir *et al.* 2013). Based on these reports, 10 samples were removed due to low technical quality. Variant calls were filtered with VCFtools (Danecek *et al.* 2011) to remove sites with quality score below 30 and read depth lower than 5. The entire variant position was removed if it contained non-SNP variants, non-biallelic variants, or had more than 33% missing data. The final high-quality dataset contained 81,301 SNPs (File S3). The genotype calling workflow was parallelized using workflow management software STAPLER (Tyrmi 2018).

### Diversity and population structure

To estimate the levels of genetic diversity, pairwise nucleotide diversity (Nei and Li 1979) was calculated with a modified version of python script provided in Garner *et al.* (2016) (File S3). The size of available genome used for analysis was 3.8 Mbp. To calculate Tajima’s D and pairwise ST the SNP data set was filtered with vcftools–thin parameter to remove variants closer than 10 kbp from each other to reduce correlation between sites due to physical proximity. After filtering, a set of 4,874 SNPs were available.

Tajima’s D estimates (Tajima 1989) were calculated for the whole dataset and also for each population separately using ∂a∂i (Gutenkunst *et al.* 2009). The allele frequency spectrum was generated using ∂a∂i with first down-projecting the sample size to 86 to account for missing data. Hudson *et al.* (1992) pairwise F_ST_ values were then calculated for each population pair by using the equation presented – and recommended over the Weir and Cockerham estimate (Weir and Cockerham 1984) – in Bhatia *et al.* (2013) for a two-population, bi-allelic scenario. An unbiased genome-wide estimate of F_ST_ for each population pair was obtained by calculating the nominator and denominator of equation 10 presented in Bhatia *et al.* (2013) separately for each site, averaged over all sites after which the division was performed.

Population structure was also examined with principal component analysis (PCA) (McVean 2009) using the prcomp R package. Sample size was evened between different populations to six as uneven sample sizes may distort the PCA projections. For the analysis singleton variants were removed as recommended for instance by Galinsky *et al.* (2016).

Population structure was further analyzed by using STRUCTURE software (Pritchard *et al.* 2000). As STRUCTURE is computationally demanding the SNP set was stringently filtered to obtain a smaller high-quality data set of 4,197 bi-allelic SNPs. At least 10 kbp distance between variants, minor allele frequency of 0.3 and maximum proportion of missing data per site of 0.2 were used. Burn-in length of 250,000 and run length of 50,000 steps were used. K values from 1 to 10 were tested with three replicate runs for each value of K. The software package Clumpak (Kopelman *et al.* 2015) was used to visualize the results and in determining the most likely value of K by using the method of Evanno *et al.* (2005).

The R-package conStruct (Bradburd *et al.* 2018) was used for spatial analysis of population structure. It allows explicit testing for presence of isolation-by-distance, often found in continuous populations and thus reduces the probability of overestimating the number of potential clusters. Two models, non-spatial which is similar to the model the ADMIXTURE software uses (Alexander *et al.* 2009), and a spatial model which accounts for isolation-by-distance patterns, were tested. For both models K-values from 1 to 5 were examined using 50,000 iterations in each. To test whether the spatial or non-spatial model better explains the genetic variation and to compare results between different K-values the cross-validation pipeline provided with conStruct was used with 50 iterations up to K value of 8 (Figure S2).

The level of LD in the dataset was estimated by nonlinear regression of *r^2^* on between-site distance in base pairs (Hill and Robertson 1968). The expected relationship is presented in the following equation, $E\left(r^{2}\right)=\left[\frac{10+\rho d}{\left(2+\rho d)(11+\rho d\right)}\right]\left[1+\frac{\left(3+\rho d\right)\left(12+12\rho d+\rho^{2}d^{2}\right)}{n\left(2+\rho d\right)\left(11+\rho d\right)}\right]$, where n is the sample size, d is a distance between adjacent sites in base pairs, $\rho =4N_{e}c$, population recombination rate between adjacent sites and *c* is the recombination rate (Hill and Weir 1988). *r^2^* was calculated between all variants located within the same scaffold over all populations as the populations are nearly panmictic according to the conStruct analysis. Singletons were omitted from this analysis.

To detect loci forming allele frequency clines along the sampled latitudinal gradients, possibly indicating that they are under varying selective pressure along the gradient, a test of generalized linear mixed effect models was fitted for all loci using R package lme4. The first model was created with glmer function for each SNP by setting genotypes as a response variable, population information as a fixed effect and latitude as a random effect. The second model was created similarly but with latitude omitted. The two models were then compared to each other by calculating a p-value with ANOVA to infer whether or not latitude contributes to the model. In addition to latitude we also performed the analysis using 21 other environmental variables downloaded from WorldClim (Hijmans *et al.* 2005). The Baza population was omitted from all selection scan analyses as it was shown to be the only population clearly differentiated from the others in every analysis of population structure.

To identify putative loci responsible for local adaptation we used the program pcadapt (Luu *et al.* 2017). It infers population structure with PCA and then identifies putative outliers with respect to how they are related to the population structure, making it well suited for examining datasets containing isolation-by-distance patterns. We used all SNPs with minor allele count over 10 and with Baza population omitted for generating PCA. The number of principal components to be used in the outlier analysis was chosen as two, by first producing a scree plot (Figure S3) with pcadapt and then applying Cattell’s graphical rule. Pcadapt assigns a p-value for each SNP. The p-value distribution (Figure S4) is then used to obtain FDR estimates.

To further detect potential loci underlying local adaptation, we also used the Bayesian F_ST_-outlier method bayescan (Foll and Gaggiotti 2008) that is based on identifying locus specific components affecting allele frequencies as a signal of selection. Bayescan was run with default parameters with the exception of setting prior odds for the neutral model to 100 from the default of 10 to account for the large set of SNPs as recommended in the bayescan documentation.

Bayescan analysis also revealed the presence of a large haplotype structure in 11 samples with SNPs in complete LD in several scaffolds. To find all scaffolds included in the haplotype structure an r^2^ value was calculated between one of the SNPs contained in the haplotype and all other SNPs in our dataset. Scaffolds containing one or more SNPs with *r^2^* value of 1.0 in this comparison were then assumed to be part of the haplotype structure. As it is possible that SNPs are in complete LD only by chance, we examined how likely it is that the whole haplotype structure would be due to chance. This was done by randomly choosing 10,000 sets of scaffolds with each set having similar properties to the ones containing the haplotypes and testing how often similar haplotype structure could be seen. More specifically, each randomized set contained similar number of scaffolds (59) with similar number of SNPs (25 per scaffold) and contained at least one SNP with equally high or higher minor allele frequency (11/109) as the scaffolds containing the haplotype. The permutation approach was also used to further study if the haplotype had been affected by selection by calculating within population π and between population F_ST_ and d_XY_ values for the permuted set. F_ST_ is a commonly used measure for differentiation, but it can be affected by reduced within-population nucleotide diversities. d_XY_ is a measure for estimating absolute levels of differentiation and is unaffected by this potential bias, although it is susceptible to bias stemming from unequal sample sizes (Nei and Li 1979; Cruickshank and Hahn 2014). F_ST_ values were calculated as described earlier, d_XY_ was calculated using the following equation: $d_{xy}=\;\frac{1}{n}\overset{n}{\underset{i=1}{\sum }}p_{i1}\left(1-p_{i2}\right)+p_{i2}(1-p_{i1})$. Results of the permutation analysis were then compared to observed values of the haplotype region.

### Data availability

Figure S1 contains p-value distributions for linear regression analysis. Figure S2 contains cross-validation results for conStruct analysis. Figure S3 contains scree plot for pcadapt analysis. Figure S4 contains p-value distribution for pcadapt analysis. Figure S5 contains between-population covariance visualizations for conStruct analysis. Figure S6, S7 and S8 contain boxplot representation for between population F_ST,_ d_XY_ and pairwise within population π for the peculiar haplotype pattern region and for permuted datasets. Table S1 lists interesting outliers for linear regression analysis. Table S2 shows pairwise F_ST_-values for all population pairs with the putative inversion removed. File S1 contains the final 60,000 bait set sequences in fasta format. File S2 contains additional method description for filtering paralogous variants. File S3 contains SNP-data in vcf-format used in the analysis of this publication. Custom script used in calculating π is provided in File S4. Raw Illumina sequences are available at NCBI SRA with accession number PRJNA592869. Supplemental material available at figshare: https://doi.org/10.25387/g3.12403448.

## Results

### Nucleotide diversity

**Figure 2:**
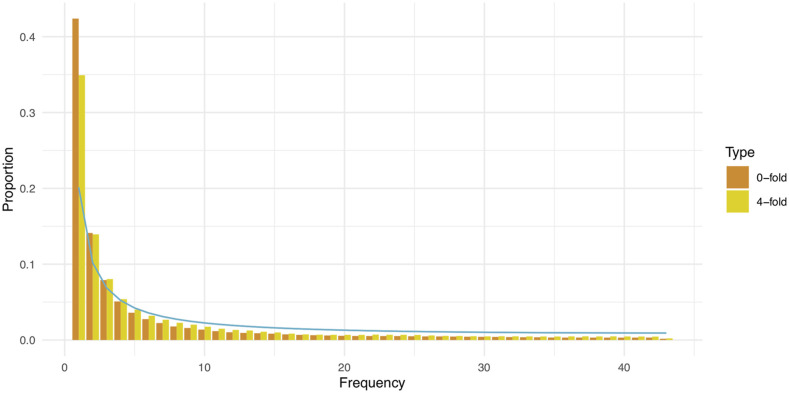
Minor allele frequency spectrum calculated over all samples. Spectrum is projected down to 87 samples to account for missing data. Blue line denotes expected spectrum shape calculated according to equation presented in *Nordborg et al. (2005)* figure 7.

Pairwise synonymous nucleotide diversity averaged over populations was 0.0039 with different populations showing similar diversity (Table 1). $\pi_{N}$/$\pi_{S}$ ratio was on average 0.394 and again similar levels can be seen in all populations indicating homogenous levels of negative selection across populations. Tajima’s D value over all populations was -1.29 and it was also negative within every sampled population with Baza population having a less negative value than others (Table 1). This result is also reflected in the minor allele frequency spectrum calculated for all samples (Figure 2), which shows an excess of rare alleles compared to the standard neutral expectation.

### Assessing population structure

We performed a pairwise F_ST_ (Hudson *et al.* 1992), STRUCTURE (Pritchard *et al.* 2000) and PCA (McVean 2009) analysis to evaluate the genetic relationships between populations. All analysis indicate that the Spanish Baza population is differentiated from other populations and in addition to that, very subtle population structure separates eastern and western samples from each other. In general, pairwise F_ST_ estimates show low level of differentiation between most sample populations (Table 2) with overall F_ST_ of 0.031. Contrasts with Baza are higher (average of 0.079).

**Table 2:** Weighted genome-wide averages of pairwise F_ST_ estimates for all populations

Population	Inari	Kolari	Kälviä	Punkaharju	Kalsnava	Radom	Ust-Chilma	Megdurechensk	Ust-Kulom	Penzenskaja	Volgogradskaja
**Kolari**	0.013										
**Kälviä**	−0.001	0.016									
**Punkaharju**	0.000	0.017	0.005								
**Kalsnava**	0.019	0.019	0.023	0.020							
**Radom**	0.002	0.021	0.005	0.007	0.015						
**Ust-Chilma**	0.010	0.022	0.013	0.010	0.034	0.018					
**Megdurechensk**	0.008	0.021	0.011	0.009	0.029	0.016	0.001				
**Ust-Kulom**	0.064	0.045	0.063	0.064	0.044	0.052	0.054	0.052			
**Penzenskaja**	0.009	0.023	0.011	0.013	0.033	0.015	0.008	0.009	0.060		
**Volgogradskaja**	0.000	0.017	0.005	0.005	0.023	0.007	0.010	0.006	0.063	0.000	
**Baza**	0.068	0.082	0.073	0.071	0.077	0.065	0.083	0.077	0.117	0.081	0.072

**Figure 3:**
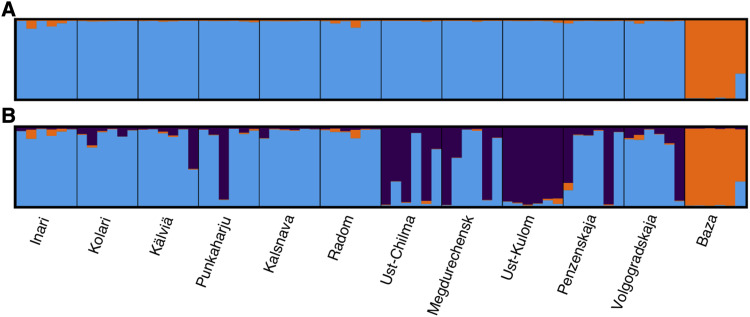
Visualization of STRUCTURE results using K values of 2 (A) and 3 (B).

**Figure 4:**
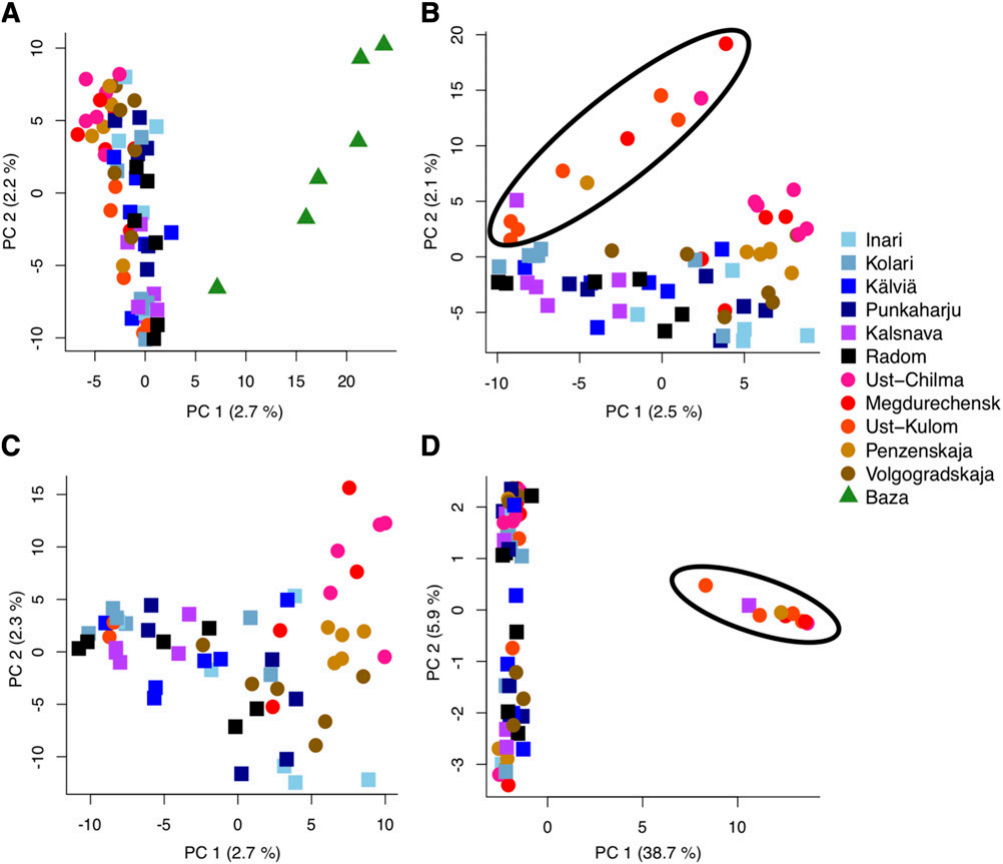
PCA projections of two first principal components of all samples (A), excluding Baza population samples (B), excluding Baza and the samples containing the putative inversion (C) and projection created using variants from putatively inverted area (D). In figures B and D the samples encompassed within black circle contain the putatively inverted haplotype. Circles represent the samples of the western cline, squares the samples of eastern cline and triangles the samples in isolated Baza population. Total variance explained by principal component is indicated within parentheses next to respective principal component axis header.

The STRUCTURE results (Figure 3) analyzed using Evanno method indicate that the most likely value of K is three. Again, Baza population forms a distinct group compared to the main distribution. However, the rest of the range is divided into two groups, where the other contains all samples of Ust-Kulom population and parts of other, mainly eastern, populations. PCA analysis suggests that the genetic differentiation within and across populations in general is weak as each principal component explains just small fraction of total variance (Figure 4A), although some trends can be observed. The samples originating from Baza population are separated from the rest by the first principal component. The rest of the range is being clustered more closely together, with a trend separating the eastern and western samples from each other (Figure 4B, 4C).

**Figure 5:**
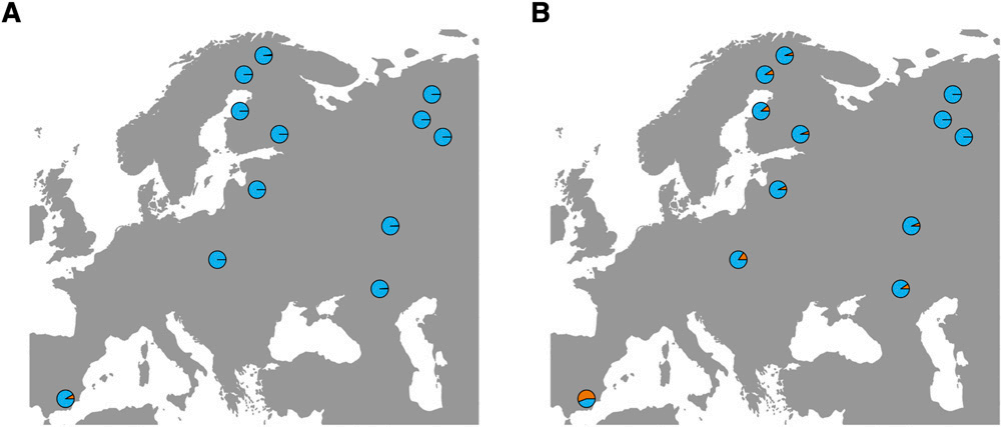
Admixture proportions for two layers estimated for different populations using conStruct spatial (A) and non-spatial (B) models.

ConStruct analysis was performed using the both non-spatial model and the spatial model (Figure 5), which incorporated information on the geographical distance between populations. A cross-validation test indicated that for the spatial and non-spatial models the predictive accuracy improved with more layers, but only modest improvement can be seen after K2 (Figure S2). At K2 the spatial model has a better fit than the non-spatial model. Even though the K2 model has two layers, the second layer contributes very little (1–2%) to populations other than Baza where it contributes 8% (Figure 5A). We also inspected the value of parameter $\alpha_{D}$ which controls the shape of the decay of covariance in the spatial model, with values close to 0 indicating no isolation-by-distance (equation 3 in Bradburd *et al.*, 2018). The first layer with larger proportion produces a value of 0.0020 for $\alpha_{D}$ indicating that a very weak isolation-by-distance-pattern can be detected through most of the sampled distribution. Other layer parameters describing the isolation-by-distance control for the sill of covariance matrix in each layer ($\alpha_{0}$= 0.0098), control the shape of the decay of covariance with distance in each layer ($\alpha_{2}$ = 0.093), global variance due to shared ancestral frequency (γ=0.144) and population specific drift parameters (*i.e.*, nugget values) (0.051-0.058) (Figure S5A for K1 and Figure S5B for K2). Most of the covariance shown in the figure S5B (0.185) is explained by the covariance originating from the same ancestry (*i.e.*, layer). The within population covariance (dots) is slightly higher by 0.06. The contribution of IBD is very small but nonetheless explains the data better than a spatial model without the alpha parameters.

### Identifying loci responsible for local adaptation

Comparison of linear regression models with or without latitude was used to identify clinal allele frequency patterns. At a p-value cutoff of 0.001 a total of 12 SNPs were outliers, although due to limitations in the annotation of the reference genome the putative gene of interest could be defined only in three cases. Outliers that could be annotated were the Early-responsive to dehydration stress 1 (ERD1) known to play a role in dehydration stress (Simpson *et al.* 2003), a putative pentatricopeptide repeat containing gene and an ATP−binding cassette transporter gene. With a more lenient p-value cut-off of 0.01 a total of 131 SNPs were outliers, including several genes with interesting function (Table S1). However, controlling for false discovery rate (FDR, Benjamini and Hochberg 1995) with q-values obtained from the p-value distribution suggests that a high proportion of top candidates are false positives as the minimum q-value for the dataset was 0.46. The sampling was designed to particularly detect latitudinal allele frequency clines, but we also downloaded all available environmental variables for each sampling site from WorldClim and performed the same analysis, yielding no outliers with low q-values as with the tests using latitude. The lowest q-value of 0.18 for a top outlier SNP was obtained with longitude. Interestingly, the top outliers seem to have identical allele frequencies population-wise with high minor allele frequency especially in the Ust-Kulom population. These SNPs, although still suggesting a high chance for type I error, were further examined by studying their LD patterns. Other environmental variables also yielded even p-value distributions (Figure S1) suggesting few true outliers.

**Figure 6:**
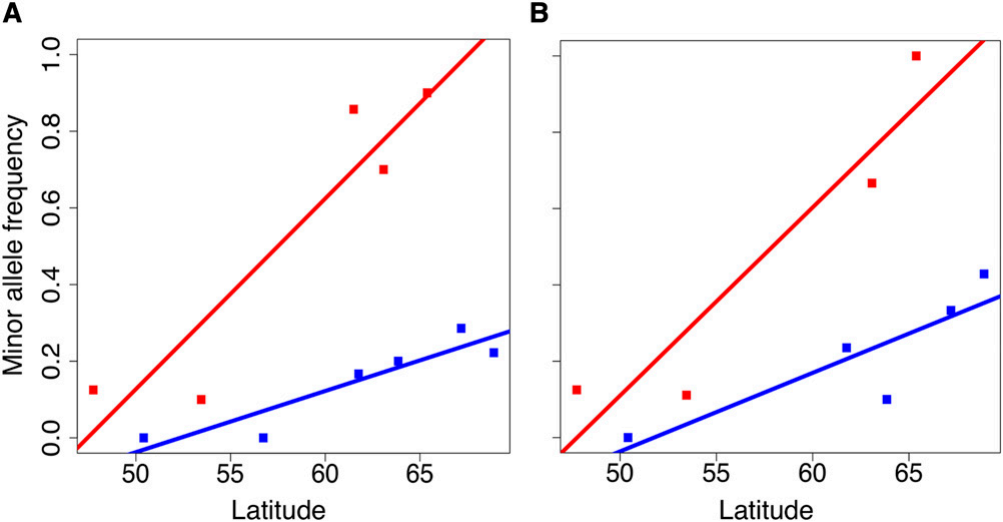
A) Bayescan outlier locus allele frequencies at sampling sites (Y-axis) across latitude of the sites (X-axis). Populations are marked with red (eastern) and blue (western) squares with respective least squares trend line. B) Allele frequency of the second highest scoring bayescan result.

Bayescan, an F_ST_ outlier detection method, was used to detect putative SNPs underlying local adaptation. Using 0.1 FDR level we obtained a single outlier locus, which is located in non-coding area of the *P. taeda* reference genome v. 1.01 in position 404,961 of tscaffold3905. TreeGenes database (Wegrzyn *et al.* 2008) *P. taeda* annotation also places the area into non-coding area, but blast search of the surrounding sequence against all known gymnosperm genes at ConGenIE (http://congenie.org/) (Sundell *et al.* 2015) revealed that the outlier locus appears to lie within a gene with an unknown function. The outlier locus has a distinct allele frequency pattern (Figure 6A) where the frequency of the alleles varies along latitude with steeper cline in the east. Interestingly, the second highest scoring variant, although above the 0.1 FDR limit, shows a very similar allele frequency cline pattern to the first outlier (Figure 6B). The variant is located within an intron of a Rubisco gene family member. The two top variants are not in LD nor do they appear to be located in the same scaffold of the *P. taeda* reference genome. The subsequent SNPs in p-value rank, although also above the FDR limit, are the same set of SNPs detected in linear regression analysis with identical allele frequencies.

The pcadapt scan (Luu *et al.* 2017), used for identifying loci under selection by searching for excess divergence along principal components of population structure, assigned a total of 489 SNPs as outliers when a q-value cutoff of 0.1 was used. This set also includes the set of SNPs with identical allele frequencies detected in Bayescan and the linear regression analysis.

### Linkage disequilibrium patterns

**Figure 7:**
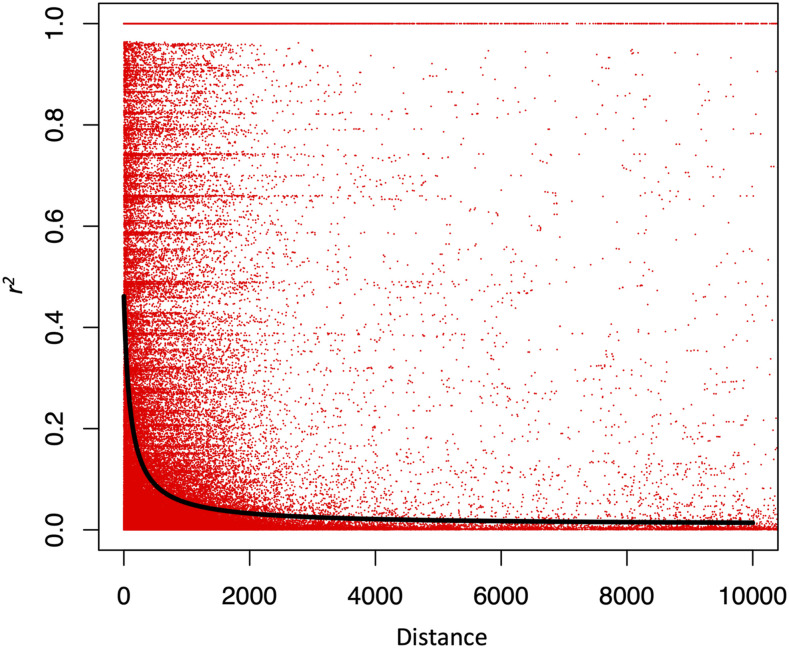
Linkage disequilibrium coefficients (*r^2^*) based on all pairwise SNP comparisons for all samples. Black line shows the squared correlation of allele frequencies *r^2^* against physical distance between the SNPs (Hill and Weir 1988).

Linkage disequilibrium patterns (Figure 7) suggest that LD decays quickly within the *P. sylvestris* genome as the $r^{2}$ values fall below 0.2 within 145 bp. However, outlier scans revealed that many SNPs in different scaffolds had identical allele frequencies within each population, with particularly high minor allele frequency in the Ust-Kulom population. More careful examination of the LD pattern for these SNPs revealed a distinct haplotype structure in 11 samples, of which five belong to the eastern Ust-Kulom, two to Megdurechensk, two to Penzenskaja, one to Ust-Chilma and one to the Latvian Kalsnava population. We did not find any putative technical explanations for the phenomenon, as stringent parameters were used in the read alignment and SNP calling, where only concordantly, uniquely aligned reads were retained. In addition, the read alignments were visually inspected using SAMtools tview, and no abnormalities were found.

**Figure 8:**
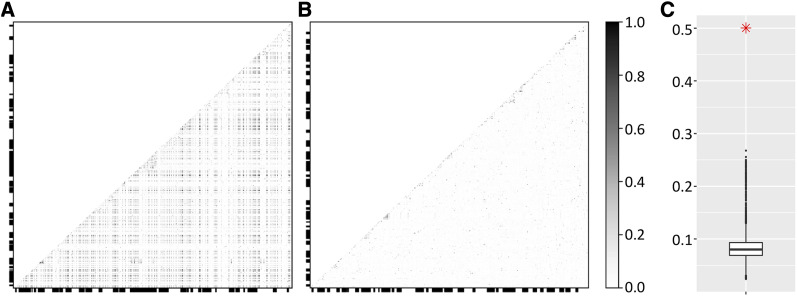
A) Heatmap visualization of allelic correlation coefficient $\left(r^{2}\right)$ values below diagonal calculated between all SNPs identified as being part of an inversion, and all variants within their surrounding 1 kbp areas. Alternating thick and thin X and Y axis borders denotes variants belonging to the same scaffolds. B) Similar heatmap to A, but random variants with similar allele frequency to the inversion were selected along with their 1 kbp surrounding areas to visualize typical linkage disequilibrium patterns. Some scaffolds show LD within them, but between scaffolds mostly only low values $r^{2}$ can be seen. C) Means of $r^{2}$ values for 10,000 random 1kb areas (one of which is visualized in 1B heatmap) marked in black and the mean $r^{2}$ value of blocks containing the putative inversion haplotype marked with red asterisk.

In total 169 variants located in 59 different reference sequence scaffolds had identical allele frequency and LD pattern (Figure 8A). The possibility of detecting such haplotype structure by chance was explored using a permutation test showing that the probability is very low (p-value < 0.001). In the set of samples exhibiting the haplotype structure, the average nucleotide diversity within the 1-kbp area surrounding each outlier variant was only 0.0003, compared to value of 0.0034 within the same region observed between other samples. Average pairwise nucleotide diversity value calculated between the samples exhibiting the haplotype structure and other samples was 0.0093 for the same area consistent with higher than average F_ST_ values. Variants were found to be polymorphic only within the haplotypes, but no variant was polymorphic within both haplotypes, providing further proof that no recombination events between them have taken place.

Westbrook *et al.* (2015) generated a consensus genetic map for *P. taeda* and aligned many of their EST sequences corresponding to the marker data to the *P. taeda* reference genome v. 1.01, thus providing putative physical location for many scaffolds of the reference. There were 12 cases where the EST sequence alignment covers or is within a few kbp of the SNPs belonging to the *P. sylvestris* haplotype structure. In ten of these cases the Westbrook *et al.* (2015) data suggests that the scaffolds belong to linkage group one, but one scaffold seems to be part of linkage group 3 and another on of linkage group 10. The SNPs positioned in linkage group 1 are located between positions 51.12 cM and 94.54 cM. This 43.42 cM area is 23.48% of the total length of the first linkage group. Given that the 12 chromosomes of *P. sylvestris* seem to be similar in size, we can expect each chromosome to be roughly 2.0 Gbp long. We can then, naively, take the proportion the haplotype structure covering the first chromosome’s genetic map and apply it to the expected physical size of the chromosome. This approach gives us an estimated size of 470 Mbp for the haplotype structure. Another way for estimating the haplotypes minimum size is to simply sum the lengths of the *P. taeda* reference scaffolds containing the haplotype structure, which gives a size estimate of 35.9 Mbp. As we have a capture target in 11.0% of *P. taeda* reference genome scaffolds, we estimate that the total area covered by the haplotype structure is roughly 35.9 / 0.11 = 326 Mbp.

Allele frequencies of the haplotype structure were highly similar to the detected population structure patterns. To investigate whether the haplotype drove the observed population structure, STRUCTURE, PCA and pairwise F_ST_ analysis were redone without the scaffolds containing the haplotype structure. No change was observed in the STRUCTURE results. However, the F_ST_ results appear to be affected such that lower values are now seen between Ust-Kulom and other populations (Table S2), but the values are still relatively high. It may be that several scaffolds that are part of the inversion are not identified as part of it, as they happen to not contain any informative SNPs for identifying the inversion. In the PCA results, the projection where Baza samples are omitted (Figure 4B), the samples containing the haplotype structure are separated by the second principal component and the other eastern samples are separated from western samples by the first principal component. Removing also the samples that contain the haplotype structure results in projection where Eastern and Western samples are more distinctly separated and occupy the opposing ends of the first principal component. When PCA was performed using only the areas identified as part of alternate haplotype, the samples containing the alternate haplotype structure form a distinct group with the separating principal component explaining large proportion of the variance (Figure 4D).

Permutation test was performed to test whether the patterns of between population F_ST_, d_XY_ and within population π in the haplotype region are consistent with ongoing positive selection in a subset of populations. Pairwise F_ST_ and d_XY_ values were significantly higher in comparisons between Ust-Kulom and other populations within the haplotype area compared to the permuted data (Figures S6 and S7). Values of π in the Ust-Kulom population were very low between the five samples containing the haplotype structure (0.0006). However, the values between the five samples containing the haplotype and the two samples not containing the haplotype were particularly high (0.0061), as one might expect to observe between diverged haplotypes. This resulted in mean value of π within Ust-Kulom population to be on a similar level to other populations (Figure S8). In other populations where the haplotype pattern was detected in low frequency, the mean π values were particularly high in the affected area due to the high number of pairwise comparisons between the peculiar and normal haplotypes.

## Discussion

This first genome-wide analysis of *P. sylvestris* genetic diversity covering a large proportion of its distribution show estimates of genetic diversity and population structure largely in line with previous studies. Outlier scans used for uncovering loci contributing to local adaptation detected many candidate genes, of which many have been shown to be targets of selection in previous studies (Table S1) for example in *A. thaliana* (Knoth and Eulgem 2008) and Eucalyptus (Jordan *et al.* 2017) even though such coincidence does not verify the effect of natural selection in these loci. Furthermore, a previously unidentified large structural variation, possibly related to local adaptation, was uncovered.

### Indications of population structure

The neutral nucleotide diversity found in this study is similar to what has been observed in previous studies of this species (Kujala and Savolainen 2012) and in several other conifers (Brown *et al.* 2004; Eckert *et al.* 2013; Grivet *et al.* 2017). Given the mostly continuous distribution of wind-pollinated *P. sylvestris* and the previous findings of near-absent population structure, only very negligible differences between populations were anticipated, with the exceptions of geographically isolated populations (Pyhäjärvi *et al.* 2007; Wachowiak *et al.* 2009; Kujala and Savolainen 2012). It has been suggested that putative *P. sylvestris* refugia during the last glaciation has existed in the Mediterranean area, northern Europe and also in the east, possibly in Ural Mountains (Naydenov *et al.* 2007). Therefore, weak genetic structure resulting from expansion from two distinct refugia, from east and west, was expected.

All approaches of population structure analysis uniformly indicated that the Baza population, geographically isolated from the main distribution, was most, although still weakly differentiated from the other populations. STRUCTURE and PCA analysis also gave some indication that the most likely number of groups is three. However, the models of these frequently used methods do not explicitly account for geographic isolation-by-distance, which can be assumed to exist within the distribution of many species, including *P. sylvestris*. Omission of this phenomenon from the models may cause these methods to spuriously assign populations to separate groups, when the genetic variation could in fact be explained by continuous isolation-by-distance (Bradburd *et al.* 2018). This also seems to have happened with our *P. sylvestris* analysis, where STRUCTURE suggested three distinct clusters, but the conStruct spatial model explains the vast majority of genetic covariance by within-sampling-location effect accompanied with weak isolation-by-distance pattern across populations.

Our results are in contrast with results from many other tree species, such as *Picea abies* where considerable structure has been detected despite many similarities in distribution, population size and reproductive biology (Chen *et al.* 2019). Several studies in *Populus* have also suggested the presence of distinct population structure (Keller *et al.* 2010; Evans *et al.* 2014; Geraldes *et al.* 2014). *P. sylvestris* rarely hybridizes with other species, and is not capable of clonal reproduction, but the exact connection between these characteristics and lack of major population structure is not understood. This lack of genome-wide structure is an advantage when investigating the genetic basis of adaptation, as such structure is a complicating factor in selection scans (Hoban *et al.* 2016).

### Putative signs of local adaptation

As *P. sylvestris* is known to be locally adapted to various environmental conditions within its vast distribution (Savolainen *et al.* 2007), we anticipated to identify signs of natural selection in the genomic variation. We performed an F_ST_ -based outlier scan, which identified only single statistically significant outlier SNP with an allele frequency cline in western transect and a particularly strong cline in the eastern transect, with another non-significant variant exhibiting very similar allele frequency cline to the top outlier. The first outlier did not have any reliable annotation, but the second variant was located within an intron of a Rubisco gene, which has been suggested to have a role in ecological adaptation to different temperatures and CO_2_ concentrations (Hermida-Carrera *et al.* 2017). A large number of outliers in F_ST_ based selection scan would have been unexpected considering earlier findings (Kujala and Savolainen 2012) and most theory suggesting that the nature of the underlying genetic architecture is likely highly polygenic, but observing only single outlier is surprising. It is possible that higher number of populations and samples for bayescan analysis would have allowed more outliers to be uncovered or that most of the adaptive variation is in the non-coding regulatory regions not investigated here.

We also applied the *pcadapt* method, which accounts for the possible population structure via principal component analysis and identifies outliers relative to this structure and is well suited for scenarios involving continuous population structure (Forester *et al.* 2018). The approach yielded 489 putative outliers. As expected, the outliers also included the SNPs identified as part of the haplotype structure discussed below. These SNPs were also top outliers in the linear regression analysis of allele frequencies, which also identified some other interesting genes. However, low q-values suggest low probability of true positive outliers in this test.

Several putative explanations exist for detecting low number of outliers in the bayescan and linear regression outlier analysis. First, as the targeted sequencing approach by definition will only allow examination of very small proportion of the genome, much of the adaptive variation cannot be detected. In species with large genomes, proportionately more adaptive variation is expected to be found outside coding region (Mei *et al.* 2018), which may also explain the lack of adaptive signal in the data obtained by exome capture. Second, as discussed above, it is possible that the eastern and western parts of our sampling have in fact originated from different refugia after the most recent glacial period, or several periods as the same refugia may have existed during many or most such periods. Therefore distinctive genetic adaptations may have evolved within each refugium, as suggested for instance by Naydenov *et al.* (2007), rendering in particular landscape genetics approaches ineffective and requiring larger amount of populations sampled from both east and west. Third, as the genetic basis of local adaptation has shown to be mostly polygenic, only a small proportion of all variants can be expected to be under strong enough selection for prolonged period to be detected. Detection of alleles with small effect may require considerably larger sample size. Powerful approaches exist for exploiting very large numbers of samples (Berg and Coop 2014; Field *et al.* 2016; Racimo *et al.* 2018), but even when applying such methods it may be challenging to control for population structure to avoid false positive signal (Berg *et al.* 2019).

### Linkage disequilibrium patterns and putative large inversion

Earlier work has shown that LD decays very rapidly within the *P. sylvestris* genome (Dvornyk *et al.* 2002; Pyhäjärvi *et al.* 2007; Wachowiak *et al.* 2009) and also in other conifers such as *P. taeda* (Lu *et al.* 2016; Acosta *et al.* 2019). This study allows for examining longer range patterns of LD than before as in many cases multiple target sequences are positioned within the same scaffold. Our findings are in line with the previous studies showing that $r^{2}$ fall below 0.2 within 145 bp. An advantage of low the level of genome-wide LD observed previously, and in this work, is that variants detected in the outlier scans are probably very close to the causative polymorphism (Neale and Savolainen 2004).

Interestingly, the linear regression, bayescan and PCAdapt analysis revealed a large number of SNPs forming an unexpected haplotype structure. Permutation test shows that the LD pattern detected is not expected by chance alone. Analysis of pairwise nucleotide differences of the affected region shows that very low level of nucleotide diversity can be observed within the samples where the haplotype is present, but in the other samples the diversity level seems comparable to average genome-wide level. Simple estimates indicate that it is very likely to be several hundred million base pairs long.

Several biological explanations exist for detecting such haplotype pattern. First, they can be created by partial selective sweeps but considering that the haplotype structure spans 43 cM in the *P. taeda* genetic map, this explanation of sweep does not seem possible under the normal recombination rates. Nonetheless, this explanation cannot be completely dismissed. Extended haplotype patterns may also be caused by natural recombination rate variation along the genome, but this explanation is unlikely in this case, as the haplotype is strictly limited only to specific samples and geographically to the east and it seems unlikely that local recombination rate would be such different only in a subset of individuals. Also, the fact that LD is complete suggests that no recombination events have occurred between the haplotypes.

Considering all the observations we have made of the haplotype structure, an inversion contributing to local adaptation seems to be the most probable cause. Inversions, unlike the other putative explanations discussed above, can create large areas of restricted recombination as they prevent proper chromatid pairing (Andolfatto *et al.* 2001). Several specific methods do exist for identifying inversions (Kosugi *et al.* 2019), but to our knowledge none is applicable to our data, as they require a good quality reference genome, and preferably a whole genome sequence data set. When a complete reference genome for *P. sylvestris* becomes available, a whole genome sequencing combined with a method such as DELLY (Rausch *et al.* 2012) or GRIDSS (Cameron *et al.* 2017) could be used to confirm the existence of a structural variation. Alternatively, it might be possible to compare genetic maps generated from two crosses, with and without the inverted haplotype.

We could find no previous observations of long haplotypes in *P. sylvestris* literature, and only very few mentions of putative inversions in an earlier cytological study (Muratova 1997) in line with relatively strong synteny *e.g.*, beween *P. sylvestris* and *P. taeda* (Komulainen *et al.* 2003). Large inversions have been suggested as being targets of selection in many species (Wellenreuther and Bernatchez 2018), with the largest such inversions exceeding 200 Mbp between two *Helianthus* sister species (Barb *et al.* 2014). To our knowledge, the putative inversion detected in our study remains as the largest one to show any indication to contribute to local adaptation to date, although the relative size the inversion compared to whole genome size is certainly not as large as inversions in some other species. The samples containing the inversion polymorphism seem to be located broadly in the similar, but not identical, geographic area where STRUCTURE and PCA analysis indicate slight population structure border. This may suggest that the inversion event may have occurred within the speculated eastern refugium, but further investigation would be required to uncover the possible origin.

It is plausible that this area is an inversion undergoing rapid increase in frequency due to selection, as it was not only picked out by outlier scans, but also further evidence from the permutation analysis of pairwise F_ST_ and d_XY_ show patterns congruent with ongoing selection as extreme divergence of Ust-Kulom population (Figure S6-S7). However, further empirical studies are needed to confirm the phenotypic and fitness effects of the inversion and to examine whether the pattern is caused by recent locally adaptive allele or a global sweep that has not yet spread into lager geographic area. The ongoing spread of globally beneficial alleles may produce outliers in allele frequency based analysis (Booker *et al.* 2019). The latter scenario is not as plausible, as globally beneficial allele would likely already have spread in wider geographic area via efficient pollen flow.

Possible role of inversions in local adaptation had been recognized in genetics research early on (Dobzhansky 1970), and the concept of ‘supergenes’ has since been further explored (Thompson and Jiggins 2014). Inversions may contribute to local adaptation if they encompass more locally adapted alleles than alternate haplotypes (Kirkpatrick and Barton 2006), if the inversion contains alleles with positive epistasis (Feldman *et al.* 1996) or if there is particularly low deleterious mutation load within the inversion (Nei *et al.* 1967). If fitness advantage arises, the inversion will rise in frequency within the geographic area it provides selective advantage until it reaches migration-selection balance. Empirical studies have uncovered inversions contributing to local adaptation for instance in *D. melanogaster* (Kapun and Flatt 2018), sticklebacks (Jones *et al.* 2012), yellow monkeyflower (Gould *et al.* 2018), teosinte (Pyhäjärvi *et al.* 2013), humans (Puig *et al.* 2015) and in many others (Wellenreuther and Bernatchez 2018).

Further studies are required to examine how the haplotype structure and the other detected outliers affect fitness and if they contribute to local adaptation. Regardless of the mechanistic reason for the haplotype pattern, the existence of such geographically restricted haplotype is significant, because they have not been reported in large conifer genomes before.

## Conclusions

In this work we have examined the genetic diversity of *P. sylvestris* along a large portion of its range. Some patterns of population structure can be seen in a marginal population but within the continuous main range the isolation-by-distance explains well any differentiation detected, unlike in many other tree species. This mitigates the issues caused by structure in detecting signs of selection, but our results also show that while clear phenotypic signals of local adaptation have been detected, the molecular background remains largely elusive even if many well-established approaches were used here to detect the signature of selection. However, many interesting outliers were detected that have been shown to contribute to local adaptation in earlier studies. Furthermore, in this study we find a very large putative inversion, likely spanning an area equivalent to several *Arabidopsis thaliana* genomes. To our knowledge, this is the first time that a potentially non-neutral inversion has been shown to segregate in conifers, even though such occurrences can certainly be expected by theory and evolutionary important inversions have observed in wide set of plant species (Yeaman 2013; Huang and Rieseberg 2020).
